# Chemo-/immuno-/radiotherapy combination in treatment of solid cancer

**DOI:** 10.18632/oncotarget.27141

**Published:** 2019-09-10

**Authors:** Lukas Käsmann, Julian Taugner, Farkhad Manapov

**Affiliations:** Department of Radiation Oncology, University Hospital LMU Munich, Munich, Germany; Comprehensive Pneumology Center Munich (CPC-M), Munich, Germany; German Cancer Consortium (DKTK), Partner Site Munich, Munich, Germany

**Keywords:** multimodal treatment, radiotherapy, immune checkpoint inhibition, chemotherapy, PD-1/PD-L1

In the last years important advances in diagnosis and treatment of solid cancer have translated into dramatic changes in patient outcome. Most of this success has been reached due to immune checkpoint inhibitors (ICIs) which became an important pillar in the anticancer treatment aside from chemotherapy (CT), surgery and radiation (RT). As a result, ICIs such as cytotoxic T-lymphocyte-associated antigen 4 (CTLA4) and the programmed cell death protein 1 (PD1)/ programmed cell death ligand 1 (PD-L1) inhibitors revolutionized anticancer treatment [1–3].

In clinical studies, ICIs were associated with durable treatment response and lower rates of severe toxicity compared to conventional chemotherapy [4]. In order to find further improvements, randomized trials investigating combination of CT with ICIs in patients with metastatic disease have already revealed a significant survival benefit of multimodal systemic treatment compared to chemotherapy alone and suggested a synergistic interaction of chemo- and immunotherapy [3].

In addition, preclinical studies revealed a synergistic interaction between RT and ICIs and multimodal treatment approach has been tested in randomized clinical trials and were already practise changing [2].

For a long time, RT has been used as local treatment modality due to the radiation-induced death of tumor cells and was considered to be immunosuppressive due to the normal tissue damage of immune cells [5]. The effect of RT in the irradiated field has been used in anticancer treatment, but tumor response outside the irradiated volume has been also observed. The effect that RT can reduce tumor growth outside the irradiated field, is called the abscopal effect and could be explained by radiation-induced cancer cell death, cytokines, damage-associated molecular patterns (DAMPs), tumor- and neoantigens which are generated by RT and trigger anti-tumor immune surveillance i.e. make tumor visible for the immune system. Parallel, radiation-induced modulation of the tumor microenvironment may also facilitate the recruitment and infiltration of the effector T cells. In 2004, Demaria et al. revealed that the abscopal effect is immune-mediated [5]. In their murine model, RT alone just led to growth delay of the irradiated but had no effect on the non-irradiated tumor lesion. The combination of RT and growth factor Flt3-Ligand (Flt3-L) impaired the irradiated and non-irradiated tumors significantly. Furthermore, Flt3-L alone had no effect and T-deficient mice showed no growth delay of non-irradiated tumor.

In order to overview the current status of clinical research of immune checkpoint inhibition combined with radiotherapy/chemoradiotherapy, we recently reported the results of a German radiation oncology survey regarding clinical experience with focus on oncological benefit and treatment toxicity [1]. Fourteen different departments of radiation oncology at university hospitals were evaluated and a great acceptance of this new combined modality treatment paradigm was found. Combinations of chemoradiotherapy/radiotherapy with checkpoint inhibitors were under investigation at the majority of all participating centres (>75%) and considered to be effective or very effective by >85% of all respondents. The treatment of intracranial metastatic disease by this combination was assumed to be very effective by the majority of most respondents (61%). However, characterization of synergistic integration of ICI in the multimodal treatment approach will be a future goal in clinical oncology [6]. Therefore, several issues need be considered for future studies:

In current trials, RT is combined predominantly with anti-PD-1/PD-L1 treatment. According to the tumor entity or based on the cancer genome a different ICI is probably needed in combination with RT. Therefore, an “optimal” ICI for the combined treatment approach needs further evaluation. Until now, decision-making is based on PD-L1 expression and tumor mutational burden (TMB). The integration of more than one ICI combined with RT/CRT could be reasonable direction for further improvements.

Until now, the optimal timing of RT and ICI is unclear. Preclinical data have shown inconclusive results comparing the efficacy of pre-, post-, and concurrent radiation together with different ICI treatments [7]. However, different combinations have already changed clinical practice. In stage III NSCLC, a consolidative PDL1 inhibition after successful chemoradiotherapy resulted in a long-lasting tumor response, improved progression-free and overall survival and rapidly changed multimodal treatment paradigm [2].

The impact of dose and fractionation of RT, particularly with respect to immune activation, has not been well investigated yet. Of interest, comprehensive characterization of tumor immunogeneicity, especially its cellular components may have an impact on immune-mediated mechanisms of irradiation. Single dose irradiation with 20 Gy has resulted in no synergy with anti-CTLA4 or anti-PD-1 antibody in murine model, in contrast to hypofractionated radiotherapy with 3 × 8 Gy in combination with anti-CTLA4 or anti-PD-1 antibody due to a recruitment of dendritic cells and activation of CD8+ T cells [6]. Despite the fact, that the latest evidence suggests hypofractioned irradiation, future studies need to further evaluate the optimal dose of radiotherapy in combination with ICIs.

Not all patients respond to ICI monotherapy and identifying biomarkers to predict responders to immune checkpoint inhibition as well as combined treatments are strongly needed. Until now, PD-L1 expression of tumor cells and TMB have considered to be mostly useful to identify patients likely to benefit from ICIs, but other potential biomarkers, such as the microbiome, cellular subset changes, cytokines and epigenetic/genetic signatures, need further evaluation.

In summary, immune checkpoint inhibition has already significantly improved oncologic outcome in several cancer types and treatment combinations with RT are currently under intensive investigation. Further prospective trials will investigate optimal fractionation, timing and immune checkpoint agents, and to identify predictive biomarkers for this combined treatment approach to maximize treatment efficacy. A close collaboration between clinicians and basic scientists is needed to solve these issues.
